# Unveiling Synergistic Antioxidant Effects of Green Tea and Peppermint: Role of Polyphenol Interactions and Blend Preparation

**DOI:** 10.3390/ijms26136257

**Published:** 2025-06-28

**Authors:** Elena Kurin, Marianna Hajská, Ema Kostovčíková, Kamila Dokupilová, Pavel Mučaji, Milan Nagy, Branislav Novotný, Silvia Bittner Fialová

**Affiliations:** 1 Department of Pharmacognosy and Botany, Faculty of Pharmacy, Comenius University, Odbojárov 10, 83232 Bratislava, Slovakia; kostovcikova2@uniba.sk (E.K.); dokupilova6@uniba.sk (K.D.); mucaji@fpharm.uniba.sk (P.M.); nagy@fpharm.uniba.sk (M.N.); fialova@fpharm.uniba.sk (S.B.F.); 2 4th Department of Surgery, Faculty of Medicine, University Hospital Bratislava, Comenius University, Ružinovská 6, 82606 Bratislava, Slovakia; marianna.hajska@fmed.uniba.sk; 3 Mathematical Institute, Slovak Academy of Sciences, Štefánikova 49, 81473 Bratislava, Slovakia; branislav.novotny@mat.savba.sk

**Keywords:** green tea, peppermint tea, antioxidant, synergy, epigallocatechin gallate, quercetin, rosmarinic acid, blending

## Abstract

This study explores the antioxidant activity of green tea (*Camellia sinensis*, GT) and peppermint (*Mentha* × *piperita*, PM) infusions, individually and in combination, focusing on how preparation methods affect their efficacy. Antiradical and intracellular antioxidant activity was assessed using DPPH, ABTS, and DCF assays, alongside interaction analysis via combination index (CI) and dose reduction index (DRI). HPLC analysis determined the polyphenolic profiles of GT and PM. GT showed the strongest antioxidant activity, with the lowest IC_50_ values (4.81 µg/mL in DPPH, 2.70 µg/mL in ABTS, 3.71 µg/mL in DCF), indicating potent radical-scavenging potential. PM exhibited moderate antiradical capacity but similar intracellular activity (IC_50_ = 3.80 µg/mL). Co-maceration followed by lyophilization of GT:PM extracts led to nearly additive interactions (CI~1.0) and allowed significant dose reduction (DRI up to 4.44). In contrast, post-mixed extracts showed assay-dependent effects, including antagonism in intracellular ROS inhibition (CI = 1.83). Equimolar mixtures of model polyphenols: EGCG, quercetin, and rosmarinic acid demonstrated enhanced effects, with the strongest synergy in ternary mixtures (CI = 0.67–0.86), potentially achievable in GT:PM combinations. These findings highlight that extract preparation critically influences antioxidant efficacy, supporting co-maceration as a promising strategy for developing effective functional formulations based on plant extract combinations.

## 1. Introduction

Green tea (*Camellia sinensis* Kuntze, GT) and peppermint (*Mentha* × *piperita* L., PM) are botanically distinct plants that have been widely consumed for centuries in the form of GT and PM tea, respectively. These beverages are not only valued for their specific flavors but also for their numerous health benefits, attributed to their rich polyphenol content [1,2]. Among the various biological activities described for both plants, antioxidant properties have been studied in depth, as their role in promoting health is undeniable [3,4,5,6]. While the individual effects of GT and PM tea have been extensively studied, the potential interactions between their bioactive compounds remain unexplored.

GT is particularly rich in catechins, such as (-)-epigallocatechin gallate (EGCG), (-)-epicatechin, (-)-epicatechin gallate, and (-)-epigallocatechin [7,8,9]. Furthermore, leaves of *Camellia sinensis* contain other phenolic compounds as flavonoids and hydroxybenzoic and hydroxycinnamic acids, including gallic acid, *p*-coumaric acid, quinic acid, and caffeoylquinic acid isomers [10]. In contrast, PM leaves contain essential oils (dominated by monoterpenes and sesquiterpenes), rosmarinic acid, chlorogenic acid, and flavonoids like eriodictyol, hesperetin, luteolin, apigenin, and their glycosides [11,12].

In medicinal applications, GT is recognized as a traditional herbal medicinal product for the relief of fatigue and sensation of weakness [13]. Other biological effects of GT were studied as well, such as its anticancer, anti-diabetic, cardioprotective, and neuroprotective properties [14]. On the other hand, PM tea is recognized as a traditional herbal medicinal product for the symptomatic relief of digestive disorders such as dyspepsia and flatulence [15]. Antioxidant, anti-inflammatory, immunomodulatory, hepatoprotective, and antimicrobial activities were described for PM essential oils and extracts [12].

Food and beverage combinations, including tea blends, are nowadays studied under the concept of “food pairing,” which aims to enhance sensory appeal [16]. In this case, the combination of GT and mint is famous in the area of North Africa, where GT gun powder is prepared in combination with fresh mint leaves, known as Tuareg, Moroccan, Tunisian, or Algerian mint tea, which is served sweetened [17]. In this area, *Mentha spicata* and *Mentha viridis* are the most consumed species [18]. Combining different types of teas or teas with herbs (tea blends) can result in synergistic effects that enhance antioxidant, anti-inflammatory, anti-diabetic, and antimicrobial activities. These combinations not only improve the therapeutic potential of individual components but also contribute to balanced phytochemical composition, sensory properties, and overall consumer and economic value [19].

Given the well-documented health benefits of both herbal substances, this study aims to evaluate the combined effects of GT and PM on antioxidant activity, investigating potential synergistic interactions between their bioactive compounds. Description and evaluation of these interactions can contribute to the development of novel tea blends that maximize both sensory and health-promoting attributes, further expanding the field of functional nutrition and plant-based therapeutics.

## 2. Results

### 2.1. Antioxidant Activity and the Impact of Preparation Method on GT and PM Extracts and Mixtures

GT and PM extracts and their combinations were explored, with emphasis on how preparation methods influence radical scavenging potential. Co-maceration (“lyo; GT:PM”) versus mixing of separately prepared extracts (“mix; GT:PM”) were compared to determine how formulation affects radical scavenging activity.

The radical scavenging activity of GT, PM, their mixtures (co-macerated vs. mixed extracts), and selected polyphenols (EGCG, quercetin, rosmarinic acid) naturally occurring in the studied teas, chosen as model antioxidants was assessed using DPPH (2,2-diphenyl-1-picrylhydrazyl) and ABTS (2,2′-azino-bis (3-ethylbenzothiazoline-6-sulfonic acid)), while intracellular antioxidant effects were evaluated with DCF (dichlorofluorescein) assays (Table 1). Among samples, the strongest antioxidant activity across assays was observed in GT: IC_50_ values of 4.81 µg/mL (DPPH), 2.70 µg/mL (ABTS), and 3.71 µg/mL (DCF), respectively. Moderate activity was observed in PM, with IC_50_ values of 6.69, 9.18, and 3.80 µg/mL, respectively.

**Figure 1:**
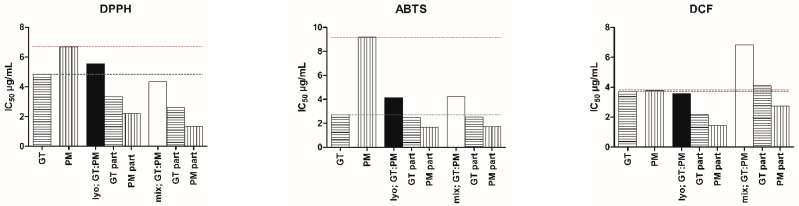
IC_50_ (µg/mL) of antioxidant activity of GT, PM, and their mixtures. Ratios show the absolute contribution (part) of each extract, showing the share of each extract in the total dose producing 50% inhibition, based on their mixing proportions. “Lyo; GT:PM” refers to co-macerated in equal proportions, lyophilized blend. “Mix; GT:PM” refers to post-mixing of individually lyophilized extracts in a 3:2 ratio based on yield. Red dotted line indicates the IC_50_ value of PM alone, green dotted line indicates the IC_50_ value of GT alone. Corresponding r-values (goodness of fit to the median-effect model) are reported in Table 1, n = 4.

As seen in Table 1 and Figure 1, some combinations appear less potent absolutely but require lower doses for a similar effect. In intracellular antioxidant DCF assay, co-macerated, lyophilized GT:PM was nearly twice as effective as the post-mixed version. When components were prepared separately, an antagonistic effect occurred. Co-maceration followed by lyophilization shifted interactions toward additivity, reducing required doses.

**Figure 2:**
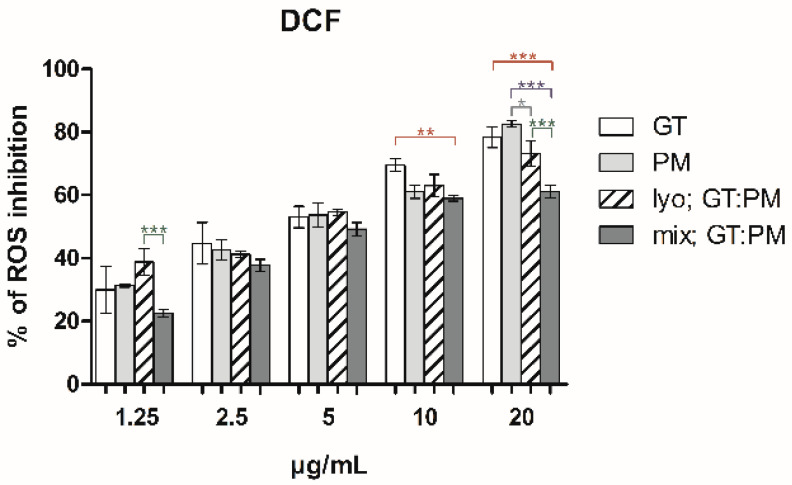
Inhibition of intracellular oxidative stress of GT, PM, and their mixtures (DCF assay on NIH-3T3 cells). “Lyo; GT:PM” refers to co-macerated in equal proportions, lyophilized blend. “Mix; GT:PM” refers to post-mixing of individually lyophilized extracts in a 3:2 ratio based on yield. The bars represent the mean ± SD, n = 4; *** = *p* < 0.001, ** = *p* < 0.01, * = *p* < 0.05 for comparisons between the samples (ANOVA/Bonferroni).

As shown in Figure 2, intracellular antioxidant effects measured by ROS inhibition in NIH-3T3 cells increased with concentration (1.25–20 µg/mL). Among samples, “mix; GT:PM” was least effective, especially at 10 and 20 µg/mL, whereas “lyo; GT:PM” and individual extracts showed higher efficacy. Rosmarinic acid, used as a positive control (IC_50_ = 1.23 µg/mL, r = 0.97), demonstrated potent intracellular antioxidant activity, which was notably comparable to that of the tested extracts.

### 2.2. Interaction Analysis of GT and PM Extracts and Mixtures

The interaction effects of GT:PM combinations were evaluated using radical scavenging assays DPPH and ABTS, and intracellular antioxidant effects measured by DCF assay, as summarized in Table 2, comparing co-lyophilized and post-mixed formulations. In the lyophilizate (“lyo; GT:PM”) prepared by co-macerating GT and PM before lyophilization, the combination index (CI) values were close to 1 in all three assays (DPPH: 0.99, ABTS: 0.99, DCF: 0.96), indicating nearly additive effects. The Dose-Reduction Index (DRI), which reflects how much the dose of each component can be reduced without loss of activity, showed varying levels of efficiency. The highest dose reductions were observed after co-maceration (“lyo; GT:PM”) in the ABTS assay (1.30:4.44), followed by DCF (1.72:2.65) and DPPH (2.42:1.74).

In contrast, the mixture (“mix; GT:PM”) made by combining separately lyophilized GT and PM showed different interaction patterns. In the DPPH assay, it exhibited slight synergism (CI = 0.80) and a more pronounced dose reduction (DRI = 3.85:1.84) than the lyophilizate. In the ABTS assay, interaction remained nearly additive (CI = 1.02) with a similar DRI trend (1.27:4.33). However, in the DCF assay, antagonism of the mixture was shown (CI = 1.83) with lower dose efficiency (DRI = 0.91:1.39) compared to the lyophilizate.

### 2.3. Antioxidant Activity of Polyphenols and Their Combinations

The radical scavenging activity of individual polyphenols–epigallocatechin gallate (EGCG), quercetin (Q), and rosmarinic acid (RA), as well as their equimolar mixtures, was evaluated using DPPH and ABTS assays. These results are summarized in Table 3. Among the individual compounds, the strongest radical scavenging activity was exhibited by EGCG, with IC_50_ values of 5.28 µM (DPPH) and 2.68 µM (ABTS), respectively. Moderate effects were observed for quercetin and rosmarinic acid, with DPPH IC_50_ values of 16.11 µM and 17.90 µM, respectively, and ABTS values of 3.36 µM and 4.44 µM, respectively. Enhanced radical scavenging capacity compared to individual compounds was observed in binary combinations. The Q + EGCG mixture was found to significantly improve activity, with IC_50_ values of 6.91 µM (DPPH) and 3.09 µM (ABTS), respectively. Similar improvements were observed in RA + EGCG and Q + RA mixtures. The most pronounced synergistic effect was displayed by the ternary combination (Q + RA + EGCG), which achieved the lowest IC_50_ values of all groups: 6.56 µM (DPPH) and 2.86 µM (ABTS), respectively.

### 2.4. Interaction Analysis of Polyphenol Combinations

**Figure 3:**
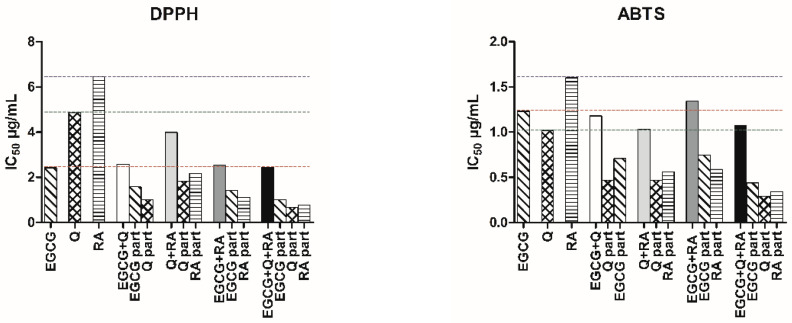
IC_50_ (µg/mL) of antioxidant activity of epigallocatechin-3-gallate (EGCG), quercetin (Q), rosmarinic acid (RA), and their equimolar mixtures. Ratios show the absolute contribution (part) of each extract, showing the share of each extract in the total dose producing 50% inhibition, based on their mixing proportions. Red dotted line indicates the IC_50_ value of EGCG alone, green dotted line indicates the IC_50_ value of Q alone, purple dotted line indicates the IC_50_ value of RA alone. Corresponding r-values (goodness of fit to the median-effect model) are reported in Table 3, n = 4.

To further explore the nature of interactions between polyphenols, combination index (CI), sequential deletion analysis (SDA), and dose reduction index (DRI) values were calculated for their equimolar mixtures at the 50% effective dose level (Table 4). Slight synergism in the DPPH assay was observed for the combination of Q + EGCG (CI = 0.87 ± 0.01), while moderate antagonism (CI = 1.40 ± 0.01) in the ABTS model was observed. Mixtures of Q + RA and EGCG + RA showed moderate synergism in DPPH (CI = 0.70 and 0.76) and slight to moderate synergy in ABTS (CI = 0.82 and 0.98). The ternary mixture (Q + RA + EGCG) exhibited the strongest synergistic effect in both assays, with CI values of 0.67 (DPPH) and 0.86 (ABTS), indicating enhanced efficacy. High DRI values confirmed that polyphenol combinations required lower doses for the same effect, demonstrating their advantage. As illustrated in Figure 3, the IC_50_ values of individual compounds were significantly higher compared to those in the mixtures, showing the enhanced antiradical efficiency achieved through their combination.

### 2.5. Phytochemical Profile of Infusions

The phytochemical analysis of PM and GT infusions revealed a complex profile of polar phenolic compounds, primarily phenolic acids and flavonoid glycosides (Table 5). These compounds were identified based on retention time (T_R_), pseudomolecular ions ([M–H]^−^), MS/MS fragmentation patterns, and comparison with available literature data, chiefly by means of databases Pubchem (https://pubchem.ncbi.nlm.nih.gov/ accessed on 3 March 2025) and MassBank (https://massbank.eu/MassBank/ accessed on 5 March 2025).

In the PM (*Mentha × piperita* L.) extract, peak 1 (T_R_ = 6.693 min) was confirmed as caffeic acid with [M–H]^−^ at *m*/*z* 179, which aligns with the identification via standard compounds. Similarly, chlorogenic acid was assigned to peak 2 (T_R_ = 9.507 min, *m*/*z* 353), with typical MS^2^ fragments such as *m*/*z* 191, confirming the esterified caffeic acid structure. Peak 4, identified as luteolin-7-diglucuronide (T_R_ = 15.807 min, *m*/*z* 637), displayed a major fragment at *m*/*z* 285, indicating the aglycone. Notably, luteolin glycosides were abundant in PM, as confirmed also by peak 8 (luteolin-7-*O*-rutinoside, *m*/*z* 593), showing characteristic fragmentation to *m*/*z* 285, consistent with the loss of a disaccharide moiety. Among all the flavonoids, this one was present in the highest amount (262.4 μg/mL). A significant compound of interest, rosmarinic acid, was found as peak 11 (T_R_ = 25.267 min) with *m*/*z* 359 and fragment ions such as *m*/*z* 197 and 161, respectively. Its quantification showed the highest concentration among phenolic acids in PM (326.0 μg/mL), confirming its dominance in Lamiaceae species [20]. Peak 6, assigned as lithospermic acid (*m*/*z* 537), exhibited fragmentation (e.g., *m*/*z* 295), in accordance with known data. This compound, structurally related to rosmarinic acid, further emphasizes the richness of cinnamic acid derivatives in PM extract. Additional phenolics such as salvianolic acids B and L (peaks 9 and 14, respectively) were also identified, both presenting [M–H]^−^ ions at *m*/*z*~717 and rich fragmentation patterns. These derivatives represent polymerized forms of caffeic acid esters, contributing to the antioxidant potential [21]. Flavonoids such as hesperidin (peak 12, *m*/*z* 609) and diosmin (peak 13, *m*/*z* 607) were also found in moderate concentrations, highlighting the diversity of glycosylated flavones.

The LC-MS analysis of GT water extract revealed a rich and diverse composition of phenolic compounds, including hydroxybenzoic acids, flavan-3-ols, and their galloylated derivatives, as well as flavonol glycosides. A total of 20 peaks were detected and identified. One of the most abundant compounds identified was epigallocatechin gallate (EGCG) (peak 13, T_R_ = 16.860 min, [M–H]^−^ = 457), a major bioactive catechin known for its antioxidant, anti-inflammatory, and chemopreventive properties [22]. The MS^2^ fragmentation yielding ions at *m*/*z* 305 and 169, respectively, confirms the presence of the epigallocatechin core and galloyl moiety, respectively. Similarly, epigallocatechin itself (peak 6, T_R_ = 12.100 min, *m*/*z* 305) was detected in notable amounts (97.2 μg/mL), indicating the prevalence of non-galloylated flavan-3-ols as well. In addition, gallocatechin gallate (GCG) and catechin gallate (CG) were identified as peaks 14 and 16, respectively, with [M–H]^−^ ions at *m*/*z* 457 and 441, respectively. These compounds contribute to the overall antioxidant potential and bitter astringent taste typical of GT infusions [23]. Theogallin (peak 1, *m*/*z* 343), a galloylquinic acid derivative, was found in high concentration (152.9 μg/mL) and is considered an important compound for cognitive benefits attributed to GT [24]. Gallic acid (peak 2, *m*/*z* 169) and 2,5-dihydroxybenzoic acid (peak 3, *m*/*z* 153) further complemented the profile of hydroxybenzoic acids. Among hydroxycinnamic acid derivatives, chlorogenic acid (peak 5, *m*/*z* 353) and cryptochlorogenic acid (peak 8) were identified, both characterized by prominent fragments at *m*/*z* 191, typical for caffeoylquinic acids. A derivative, 5-*O*-caffeoylquinic acid (peak 7), was also observed, suggesting the presence of isomeric or structurally modified caffeoylquinates. An interesting polymeric phenolic compound, monogalloyl-HHDP-glucose (peak 9, *m*/*z* 633), exhibited fragment ions at *m*/*z* 463 and 349, respectively, confirming its identity as a hydrolyzable tannin, which may contribute to the extract’s astringency and potential health benefits. Flavonol glycosides were represented by rutin (peak 17, *m*/*z* 609), kaempferol-3-rutinoside (peak 18, *m*/*z* 593), and quercetin rhamnoside (peak 19, *m*/*z* 447), all showing characteristic fragments corresponding to their aglycones (quercetin and kaempferol) at *m*/*z* 301 and 285, respectively. The detection of (+)-catechin (peak 12, *m*/*z* 289), epigallocatechin 3-*O*-(3′-*O*-methyl)gallate (peak 10), and *p*-coumaroylquinic acid (peak 11) further illustrates the metabolic diversity of GT. Although most compounds were successfully identified, peaks 15 and 20 remained unassigned, though their *m*/*z* and fragmentation patterns suggest they may represent novel or less commonly reported phenolics. Overall, the profile confirms GT as a rich source of flavan-3-ols, galloylated derivatives, and flavonol glycosides, in agreement with previous literature. The high concentration of EGCG (249.7 μg/mL) and other catechins underlines GT’s relevance as a functional beverage with potential health-promoting properties, particularly in antioxidant, cardioprotective, and neuroprotective contexts [14].

Quantitatively, rosmarinic acid and its derivatives dominated in PM extract, while theogallin and catechin derivatives were more abundant in GT. The external standards used for quantification the rosmarinic acid (at λ = 280 nm) demonstrated excellent linearity (r^2^ > 0.998), enabling reliable quantification.

In terms of total percentage content (Table 6), PM extract showed significantly higher levels of THD (18.9%), and flavonoids (2.8% at λ = 392 nm) compared to GT (THD–4.7%, flavonoids–0.5%), although GT exhibited the highest total polyphenol content (29.8%), likely due to its rich catechin profile.

## 3. Discussion

Building on our previous work focused on interactions between red wine polyphenols [25], the scope was expanded by incorporating plant extracts as complex polyphenol-rich matrices. Although the antioxidant effects of GT [26,27,28] and PM [6,29,30] have been widely examined individually, their combinations have been explored less frequently. Notably, GT combined with *Ocimum gratissimum* (1:1) showed maximal synergism [31], and a GT, honey, and citrus blend enhanced antioxidant capacity [32]. Similarly, GT and stevia mixtures and PM with *Equisetum arvense* or *Desmodium molliculum* improved antioxidant profiles [33,34]. While limited data are available regarding GT and PM combinations, Moroccan mint tea (gunpowder GT and fresh spearmint) is recognized as a well-known traditional blend [17,18]. In our study, PM was selected to reflect the Central European preference for PM.

GT showed the strongest antiradical activity, consistent with its high catechin (especially EGCG) and micronutrient content that enhance its radical-scavenging activity [26]. PM showed moderate activity, with efficacy strongly associated with total phenolic content, particularly rosmarinic acid and related compounds [35].

The DPPH assay involves hydrogen atom transfer and proton-coupled electron transfer, reacting with polyhydroxy aromatics [36,37]. DPPH serves as a model for hydrogen donation from phenols and other antiradicals, providing good correlation with antiradical capacity. ABTS^•+^ scavenging occurs through hydrogen atom transfer, single electron transfer, or predominantly through sequential proton loss electron transfer mechanisms, especially in water or alcohol, where phenols ionize to phenoxide anions that act as electron donors [38,39]. These mechanistic differences highlight assay-specific reactivity of antiradicals. In the lyophilizate (“lyo; GT:PM”) obtained through co-maceration prior to lyophilization, additive effects were observed across all three assays. Notably, the greatest dose reductions were achieved after co-maceration, particularly in the ABTS assay, followed by DCF and DPPH. It is suggested that even if absolute antioxidant activity slightly declined in some assays, co-maceration enabled more efficient utilization of bioactive compounds.

The DCF assay reflects general intracellular ROS levels, not specific reactive species. Using H_2_O_2_ as a trigger, both GT and PM reduced ROS comparably, likely via scavenging, suppression, or enhancement of endogenous defenses [40]. Preparation methods markedly influenced bioactivity in cell-based experiments; co-maceration followed by lyophilization enhanced effectiveness and reduced required doses compared to separate or post-mixed extracts, suggesting that this approach enhances both potency and contributes to optimized antioxidant performance with lower input.

This improvement is likely linked to the increased content of THD (total hydroxycinnamic derivatives), which are effective antioxidants, largely due to the number of hydroxyl groups on the aromatic ring, which stabilize phenoxy radicals formed upon oxidation by ROS through electron donation. Moreover, higher levels of total hydroxycinnamic derivatives may contribute to additional health benefits, as they can enhance LDL resistance to lipid peroxidation, protect proteins, chelate metals, scavenge ROS, and inhibit enzymes involved in oxidative stress [41].

However, the preparation method significantly influenced interaction behavior. While the post-mixed extract showed some synergism (e.g., DPPH), it also resulted in antagonism in the DCF model. In contrast, co-maceration followed by lyophilization consistently shifted interactions toward additivity, particularly in the DCF model, demonstrating its importance in optimizing antioxidant interactions and underscoring the significance of the preparation method in modulating bioactivity.

Polyphenols are well-known for their antiradical effects, functioning primarily as free radical scavengers through mechanisms such as hydrogen/electron donation, singlet oxygen quenching, and metal chelation [42]. To further explore these properties, we evaluated the antiradical activity of individual model polyphenols: EGCG, quercetin, and rosmarinic acid, as well as their equimolar mixtures using DPPH and ABTS assays. Among the tested compounds, EGCG exhibited the strongest individual antiradical activity, while the ternary combination (Q + RA + EGCG) showed the most pronounced synergistic effect, achieving the lowest IC_50_ values across both assays.

Structural features of the tested compounds may explain these results. A wide range of phenol-derived compounds, especially those with *ortho*-dihydroxyl groups or pyrogallol-type substituents, contribute to ABTS^•+^ quenching, correlating with their strong antiradical performance. Quercetin and RA meet these criteria, while EGCG’s gallate moiety provides additional ester and hydroxyl functionality, further boosting its efficacy. The presence of a carbonyl group, such as the ester group in EGCG, also enhances antiradical potential [38,43]. These structural characteristics are aligned with the compounds’ performance in both assays, confirming the important role of molecular configuration in antiradical behavior.

EGCG + Q + RA combination exhibited the strongest synergism and lowest IC_50_, often outperforming individual compounds.

HPLC confirmed the presence of EGCG and quercetin glycosides in GT, and RA in PM, indicating that the enhanced antiradical potential of their combination likely results from synergistic interactions. This supports the idea that even low-activity or low-abundance compounds (multiple conjugated phenolics, especially glucuronidated and rutinosylated flavones) in herbal extracts reflect the metabolic complexity of the plant matrices and can, through interaction, contribute to substantial bioactivities.

In summary, our findings suggest that while individual compounds such as EGCG and RA play a major role in the antioxidant activity of GT and PM extracts, the method of preparation (co-macerating vs. mixing) and the complex interplay of active components also significantly influence overall efficacy. These insights underscore the importance of considering both chemical composition and processing methods when designing plant-based antioxidant formulations, particularly those intended for intracellular action or therapeutic applications.

## 4. Materials and Methods

### 4.1. Plant Material

The plant species *Mentha × piperita* cv. ‘Perpeta’ was cultivated by the Medicinal Plants Garden–Hortus Plantarum Medicarum, Faculty of Pharmacy, Comenius University Bratislava, Slovakia (GPS coordinates: 48.142073 N, 17.188698 E). The aerial parts, including leaves, were harvested at the flowering stage in July 2023 under clear, sunny weather conditions. The plants were air-dried in the shade at room temperature (25 °C). The leaves were carefully separated from the stems and flowers. Dried leaves of *Camellia sinensis* (gunpowder green tea) were purchased from Juvamed (Rimavská Sobota, Slovakia). The plant material was identified by Silvia Bittner Fialová. The identity of the plant materials was confirmed based on anatomical and morphological parameters. Voucher specimens (Vouchers No. PM_07/2023_ZLR and No. GT-Juvamed-23) are deposited at the Department of Pharmacognosy and Botany, Comenius University Bratislava.

### 4.2. Preparation of Infusions and Lyophilizates

Water infusions were prepared from GT leaves, PM leaves, and their 1:1 (*w*/*w*) mixture (“lyo; GT:PM”) following the Czechoslovakian Pharmacopoeia, 4th edition [44]. For each infusion, 15 g of dried leaves were moistened with five times their weight in deionized water and left to soak for 15 min. Subsequently, 150 mL of boiling deionized water was added, and the mixture was heated in a water bath for 5 min, followed by cooling at room temperature for 45 min. The infusions were then filtered, frozen, and lyophilized according to the manufacturer’s instructions (SCANVAC CoolSafe^TM^, LaboGene^TM^, Lynge, Denmark). The lyophilization process was performed at −53 °C and 0.043 Pa.

The final lyophilized yields were 4.4368 g (29.6%) for GT, 3.0113 g (20.0%) for PM, and 3.6980 g (24.7%) for the “lyo; GT:PM” mixture. To evaluate potential synergistic effects, a mixture of the individual lyophilizates was subsequently prepared in a 3:2 (*w*/*w*) ratio (“mix; GT:PM”), reflecting the extraction yields of the individual extracts.

Additionally, an equimolar combination of ethanolic solutions containing the major polyphenols from each infusion was prepared. These included epigallocatechin gallate (EGCG) (≥95%; Sigma-Aldrich, St. Louis, MO, USA) and quercetin (Q) (≥95%; Sigma-Aldrich, St. Louis, MO, USA) from green tea, as well as rosmarinic acid (RA) (≥96%; Sigma-Aldrich, St. Louis, MO, USA) from PM.

### 4.3. DPPH Scavenging Assay

The DPPH radical scavenging assay was conducted following the method described by Blois [45], with minor modifications. Briefly, the stable 2,2-diphenyl-1-picrylhydrazyl (DPPH) radical (Sigma-Aldrich, St. Louis, MO, USA) was freshly dissolved in methanol. A 225 µL aliquot of DPPH solution (55 µM) was mixed with 25 µL of the test sample, which included individual lyophilizates and their mixtures dissolved in deionized water, as well as polyphenols and their mixtures dissolved in ethanol. After a 30 min incubation, absorbance changes were measured at 517 nm using a Tecan Infinite M200 microplate reader (Tecan AG, Grödig/Salzburg, Austria) in 96-well microplates (Greiner Bio-One GmbH, Frickenhausen, Germany). All measurements were performed in quadruplicate. The IC_50_ values were determined from the dose-response curves.

### 4.4. ABTS Scavenging Assay

The ABTS radical scavenging assay was conducted following the method described by Re [46], with minor modifications. Briefly, a 7 mM aqueous solution of 2,2′-azinobis-(3-ethylbenzothiazoline-6-sulfonic acid) (ABTS) (Sigma-Aldrich, St. Louis, MO, USA) was prepared and mixed in an equimolar ratio with 2.45 mM potassium persulfate (K_2_S_2_O_8_) (Sigma-Aldrich, St. Louis, MO, USA). The mixture was kept in the dark at room temperature for 24 h to allow the formation of ABTS radicals. The resulting ABTS solution was then diluted with ethanol (1.1 mL) to a final volume of 50 mL. A 2.5 µL aliquot of the test sample, including individual lyophilizates and their mixtures dissolved in deionized water, as well as polyphenols and their mixtures dissolved in ethanol, was added to 247.5 µL of the ABTS solution. Absorbance changes were recorded after 6 min at 734 nm using a Tecan Infinite M200 microplate reader (Tecan AG, Grödig/Salzburg, Austria) in 96-well microplates (Greiner Bio-One GmbH, Frickenhausen, Germany). All measurements were performed in quadruplicate. The IC_50_ values were determined from the dose-response curves.

### 4.5. Intracellular Detection of Oxidative Stress Using the DCF Assay

Intracellular oxidative stress was evaluated in NIH/3T3 cells (mouse embryonic fibroblasts) by measuring reactive oxygen species (ROS) using dichlorodihydrofluorescein diacetate (DCFH-DA) (purity ≥ 97%, Sigma-Aldrich, St. Louis, MO, USA) as a fluorescent probe, following the method described by the Miranda-Rottmann team [47], with minor modifications. NIH/3T3 cells used in this study were obtained from the Department of Pharmacology and Toxicology, Faculty of Pharmacy, Comenius University, Bratislava, Slovakia. Cells were cultured in Dulbecco’s Modified Eagle Medium (DMEM) (Sigma-Aldrich, St. Louis, MO, USA) supplemented with 10% fetal bovine serum (Sigma-Aldrich, St. Louis, MO, USA), 100 µg/mL streptomycin (Sigma-Aldrich, St. Louis, MO, USA), and 100 IU/mL penicillin (Sigma-Aldrich, St. Louis, MO, USA), and maintained at 37 °C in a humidified incubator MCO-19 AIC (UV) CO_2_ Incubator with 5% CO_2_ (Panasonic, Osaka, Japan). Cells were routinely passaged approximately twice per week. For the assay, cells were seeded at a density of 15,000 cells/100 µL per well in black 96-well microplates (Greiner Bio-One GmbH, Frickenhausen, Germany) and incubated for 24 h. Following medium replacement with serum-free DMEM, 5 µL of single lyophilized samples or their mixtures were added to the wells, followed by 5 µL of DCFH-DA (final concentration of 5 µg/mL), which was added in the laboratory under dimmed light to prevent premature oxidation of the DCFH-DA. After a 15 min incubation, hydrogen peroxide (H_2_O_2_) (Sigma-Aldrich, St. Louis, MO, USA) was added to a final concentration of 100 µM. The fluorescence intensity of the oxidized DCF product was measured using a Tecan Infinite M200 microplate reader (Tecan AG, Grödig/Salzburg, Austria) with an excitation/emission wavelength of 480/530 nm. All measurements were performed in quadruplicate. The IC_50_ values were determined from the dose-response curves.

### 4.6. Interaction Analysis/Synergy Evaluation

The quantification of interaction as a synergism or antagonism was done using the combination index (CI) for n-drug combination at x% according to Chou [48].

(1) (CI)xn=∑j=1n(D)j/(Dx)j

In Equation (1), ^n^(CI)_x_ represents the sum of the doses of n drugs that together induce x% inhibition in a combination. The denominator (D_x_) refers to the dose of drug D “alone” that inhibits the system by x%. If the CI value is equal to, less than, or greater than 1, it indicates an additive, synergistic, or antagonistic effect, respectively. Through the application of the log (CI) grading scale, Chou divides synergism and antagonism into distinct ranges of the CI, as illustrated in Table 7.

The Dose-Reduction Index (DRI) indicates the factor by which the dose of each drug in a combination can be decreased at a specified level of effect, in comparison to the doses of the individual drugs. The DRI values for each drug in an n-drug combination are provided, as shown in Equation (2).

(DRI)_1_ = (D_x_)_1_/(D_1_); (DRI)_2_ = (D_x_)_2_/(D_2_); …; etc. (2)

The numerator (D_x_) refers to the dose of drug D “alone” that inhibits the system by x%. A DRI value greater than 1 signifies a beneficial reduction in dose, with higher DRI values corresponding to greater dose reductions for a given therapeutic effect. However, this does not necessarily imply synergism. Both the CI and DRI, along with the concentration required for 50% inhibition (IC_50_), were calculated using median-effect analysis via CompuSyn software version 1.0.1. (ComboSyn, Inc., Paramus, NJ, USA).

### 4.7. Phytochemical Analysis

#### 4.7.1. LC-MS/MS-DAD Analysis

Phytochemical analysis of phenolic compounds in infusions of PM and GT, as well as their quantitative content, was performed using the method described in our previous study [49], carried out using an Agilent 1260 Infinity LC System (Agilent Technologies, Santa Clara, CA, USA), equipped with a binary pump, autosampler, column thermostat, and diode array detector (DAD), coupled to a quadrupole time-of-flight mass spectrometer (6520 Accurate-Mass QTOF) with an orthogonal electrospray ionization (ESI) source (Agilent Technologies, Santa Clara, CA, USA). Separation was performed on a Kromasil C18 column (150 mm × 4.6 mm, 5 µm; Sigma-Aldrich, Munich, Germany). The mobile phase consisted of water with acetic acid at pH 2.5 (solvent A) and acetonitrile (solvent B). The elution gradient was programmed as follows: 0 min, 5% B; 0–20 min, 5–20% B; 20–25 min, held at 20% B; 25–40 min, 20–40% B; 40–60 min, 40–50% B; 60–65 min, 50–65% B; 65–90 min, 65–100% B; held at 100% B from 90–95 min; re-equilibration to 5% B from 95–96 min and held until 115 min. The injection volume was 10 µL. Mass spectrometric detection was performed in negative ion mode. Data were acquired in full scan mode over a mass range of *m*/*z* 30–1200 with MS^2^ fragmentation for compound identification. Quantitative determination was carried out using rosmarinic acid (purity ≥ 95.5%, Sigma-Aldrich, St. Louis, MO, USA) as an external standard. Chromatograms were acquired at λ = 280 nm. The phenolic compounds present in the samples were identified based on their mass spectra and accurate *m*/*z* values by comparison with available literature sources, particularly using the PubChem (https://pubchem.ncbi.nlm.nih.gov/ accessed on 3 March 2025) and MassBank databases (https://massbank.eu/MassBank/ accessed on 5 March 2025). Quantitative analysis was carried out using rosmarinic acid as an external standard. The calibration curve was obtained by injection of known concentrations (5–500 ppm). The method was validated for linearity, sensitivity, and precision. The calibration curve exhibited high linearity. The following coefficients of determination (r^2^) and validation parameters were obtained: rosmarinic acid at 280 nm r^2^ = 0.9998, regression curve y = 259.44x – 226.01, LOD = 0.50 μg/mL, LOQ = 1.7 μg/mL.

#### 4.7.2. Spectrophotometric Analysis

Quantification of selected secondary metabolites was conducted using spectrophotometric methods in accordance with the European Pharmacopoeia 11th Edition [50]. Total hydroxycinnamic derivatives (THD) were determined by Arnow’s assay at 505 nm and expressed as rosmarinic acid. Total flavonoid content was measured by the aluminum chloride method at 392 nm (luteolin-7-*O*-glucoside) and 420 nm (quercetin). Total polyphenols and tannins were quantified using the Folin–Ciocalteu reagent at 760 nm, also expressed as rosmarinic acid. All measurements were performed in triplicate using a Genesys 6 spectrophotometer (Thermo Electron Corporation, Waltham, MA, USA), and results were calculated from calibration curves and reported as mean ± SD, relative to the dried extract.

### 4.8. Statistics

All measurements were performed in quadruplicate. r-value analysis, where the r-value represents the parameter for goodness of fit to the median-effect principle of the mass-action law, was performed using median-effect analysis and sequential deletion analysis (SDA), which involves the iterative removal of one drug dose at a time for repeated CI values calculated via CompuSyn software version 1.0.1. Statistical analysis was performed using one-way ANOVA followed by Bonferroni post hoc test. Results are expressed as mean ± SD (n = 4). Statistical significance was set as follows: *p* < 0.05 (*), *p* < 0.01 (**), and *p* < 0.001 (***).

The standard error (SE) for the IC_50_ values was calculated according to Equation (6). Effect is modeled using the logistic function (Equation (3)).

(3) $$E={\left[1+{\left(\frac{{IC}_{50}}{c}\right)}^{-p}\right]}^{-1}$$

where E is the effect, c is the concentration, and p is parameter. This relationship was then linearized as (Equation (4)).

(4) $$y=px+q,\;\mathrm{where}\;y=ln\left(\frac{1}{E}-1\right),\;x=ln(c),\;q=-pln({IC}_{50})$$

Thus

(5) $${IC}_{50}=e^{-\frac{q}{p}}$$

The standard error of IC_50_ was then calculated from covariance matrix of estimates of p and q, by usual error propagation.

(6) $${SE\left({IC}_{50}\right)}^{2}=\frac{{{IC}_{50}}^{2}{SS}_{resid}}{p^{2}(n-2)}\left(\frac{1}{n}+\frac{{\bar{y}}^{2}}{{SS}_{reg}}\right)$$

## 5. Conclusions

Polyphenols and plant extracts demonstrated strong antioxidant effects, with GT infusion and EGCG standing out as the most effective. When combined, infusions prepared from the leaves of GT and PM, as well as both extracts and pure polyphenols, often showed enhanced activity through synergistic or additive effects. The method of preparation of the mixtures played a crucial role in determining these interactions. Co-maceration followed by lyophilization led to significantly improved antioxidant potential. In particular, the DCF model revealed a shift from antagonism to additivity when the extract was prepared as a single lyophilizate rather than mixed post-lyophilization. This indicates that the bioactivity of mixtures cannot be simply predicted by the effect of individual components alone. The ternary combination of quercetin, rosmarinic acid, and EGCG provided the strongest synergistic action, supporting the idea that mechanistic complementarity enhances scavenging potential. These findings highlight the importance of not only selecting effective antioxidants but also optimizing formulation and extraction methods for maximal biological efficacy.

## Figures and Tables

**Table 1:** Inhibitory activity of GT and PM extracts and mixtures against oxidative stress.

	DPPH		ABTS		DCF	
	IC_50_ µg/mL	r	IC_50_ µg/mL	r	IC_50_ µg/mL	r
GT	4.81 ± 0.31	0.92	2.70 ± 0.23	0.98	3.71 ± 0.25	0.96
PM	6.69 ± 0.28	0.97	9.18 ± 0.65	0.98	3.80 ± 0.24	0.97
lyo; GT:PM	5.53 ± 0.30 (3.32 + 2.21)	0.96	4.14 ± 0.32 (2.48 + 1.66)	0.98	3.59 ± 0.25 (2.15 + 1.44)	0.96
mix; GT:PM	4.34 ± 0.28 (2.60 + 1.34)	0.95	4.24 ± 0.36 (2.54 + 1.70)	0.97	6.83 ± 0.49 (4.10 + 2.73)	0.96

IC_50_ values were calculated via CompuSyn software version 1.0.1. Numbers in parentheses indicate absolute contribution of each component in mixtures, showing the share of each extract in the total dose producing 50% inhibition, based on their mixing proportions. “Lyo; GT:PM” refers to co-macerated in equal proportions, lyophilized blend. “Mix; GT:PM” refers to post-mixing of individually lyophilized extracts in a 3:2 ratio based on yield. Data are presented as mean ± SE, n = 4.

**Table 2:** Interaction effect, combination index (CI), SDA, and DRI values of equimolar combinations of polyphenols at IC_50_ level.

	DPPH				ABTS				DCF			
Combination	Interaction Effect	CI	SDA	DRI GT:MP	Interaction Effect	CI	SDA	DRI GT:MP	Interaction Effect	CI	SDA	DRI GT:MP
lyo; GT:PM	Nearly additive	0.99	±0.01	2.42:1.74	Nearly additive	0.99	±0.02	1.30:4.44	Nearly additive	0.96	±0.02	1.72:2.65
mix; GT:PM	Slight synergism	0.80	±0.01	3.85:1.84	Nearly additive	1.02	±0.03	1.27:4.33	Antagonism	1.83	±0.04	0.91:1.39

CI values indicate interaction type: CI < 1—synergism, CI ≈ 1—additivity, CI > 1—antagonism. DRI represents the fold dose reduction in mixtures compared to individual use. SDA involved the stepwise removal of one compound to assess its contribution. Values were calculated using CompuSyn software version 1.0.1. “Lyo; GT:PM”: co-macerated and lyophilized together. “Mix; GT:PM”: individual extracts lyophilized separately, then combined in 3:2 ratio based on yields.

**Table 3:** Radical scavenging activity of polyphenols epigallocatechin gallate (EGCG), quercetin (Q), and rosmarinic acid (RA) and their equimolar mixtures assessed by DPPH and ABTS assays.

	DPPH			ABTS		
	IC_50_ µM	IC_50_ µg/mL	r	IC_50_ µM	IC_50_ µg/mL	r
EGCG	5.28 ± 0.31	2.42	0.97	2.68 ± 0.16	1.23	0.97
Q	16.11 ± 0.27	4.87	0.99	3.36 ± 0.19	1.02	0.98
RA	17.90 ± 0.34	6.45	0.99	4.44 ± 0.29	1.60	0.97
Q + EGCG	6.91 ± 0.38 (3.46 + 3.46)	2.58 (1.00 + 1.58)	0.97	3.09 ± 0.07 (1.55 + 1.55)	1.18 (0.47 + 0.71)	0.99
Q + RA	12.02 ± 0.28 (6.01 + 6.01)	3.99 (1.82 + 2.17)	0.99	3.12 ± 0.16 (1.56 + 1.56)	1.03 (0.47 + 0.56)	0.98
RA + EGCG	6.24 ± 0.35 (3.12 + 3.12)	2.55 (1.12 + 1.43)	0.98	3.28 ± 0.14 (1.64 + 1.64)	1.34 (0.59 + 0.75)	0.98
Q + RA + EGCG	6.56 ± 0.36 (2.19 + 2.19 + 2.19)	2.44 (0.66 + 0.78 + 1.00)	0.97	2.86 ± 0.08 (0.95 + 0.95 + 0.95)	1.07 (0.29 + 0.34 + 0.44)	0.99

IC_50_ values were calculated via CompuSyn software version 1.0.1. Numbers in parentheses indicate the absolute contribution of each component in the mixtures, showing the share of each extract in the total dose producing 50% inhibition, based on their mixing proportions. Data shown as mean ± SE, n = 4.

**Table 4:** Interaction effect, combination index (CI), SDA, and DRI values of equimolar mixtures of epigallocatechin-3-gallate (EGCG), quercetin (Q), and rosmarinic acid (RA) at the IC_50_ level.

	DPPH				ABTS			
Combinations	Interaction Effect	CI	SDA	DRI	Interaction Effect	CI	SDA	DRI
Q + EGCG	Slight synergism	0.87	±0.01	4.66:1.53	Moderate antagonism	1.40	±0.01	2.17:1.73
Q + RA	Moderate synergism	0.70	±0.003	2.68:2.98	Moderate synergism	0.82	±0.01	2.15:2.85
RA + EGCG	Moderate synergism	0.76	±0.01	5.74:1.69	Nearly additive	0.98	±0.01	2.71:1.64
Q + RA + EGCG	Synergism	0.67	±0.01	7.36:8.18:2.41	Slight synergism	0.86	±0.01	3.58:4.65:2.81

CI values indicate interaction type: CI < 1—synergism, CI ≈ 1—additivity, CI > 1—antagonism. DRI represents the fold dose reduction in mixtures compared to individual use. SDA involved stepwise removal of one compound to assess its contribution. Values were calculated using CompuSyn software version 1.0.1.

**Table 5:** Polar phenolics in PM and GT extracts: retention times (T_R_), molecular ions [M-H]¯, and MS^2^ fragments, and quantitative content (μg/mL).

Peak	T_R_ (min)	[M-H]^−^ (*m*/*z*)	MS^2^ (20 eV) (*m*/*z*)	Identified Compound	Mass Concentration ppm (μg/mL) *
**PM**					
1	6.693	179.149	135	Caffeic acid	22.7
2	9.507	353.0858	191	Chlorogenic acid	20.2
3	9.873	563.0689	436/281/237/193	4′,7-Dimethoxyflavone	35.1
4	15.807	637.1032	351/285	Luteolin-7-diglucuronide	23.6
5	16.380	459.1517	297	5-Hydroxy-4′,7-dimethoxyflavone-5-glucoside	LOQ
6	18.668	537.1056	493/295	Lithospermic acid	138.7
7	20.280	595.1680	459/287	Eriodictyol-7-*O*-rutinoside (eriocitrin)	196.6
8	20.687	593.34	285.0407	Luteolin-7-*O*-rutinoside	262.4
9	22.867	717.1491	519/439/321/295/179/135	Salvianolic acid L/B	168.5
10	24.947	577.37	269	Apigenin-7-*O*-rutinoside (isorhoifolin)	128.6
11	25.267	359.19	197/161/135	Rosmarinic acid	326.0
12	28.318	609.33	301/325/284	Hesperetin-7-*O*-rutinoside (hesperidin)	97.0
13	28.457	607.36	299.0554/284.0334	Diosmetin-7-*O*-rutinoside (diosmin)	144.1
14	29.846	717.1480	537/519/493/321/295	Salvianolic acid B/L	215.0
15	36.376	715.1307	535/293	Salvianolic acid C derivative	62.8
**GT**					
1	4.773	343.0680	191	Theogallin	152.9
2	5.513	169.0145	125/107	Gallic acid	80.1
3	8.340	153.0191	327/125/124/123/109/108	2,5-Dihydroxybenzoic acid	82.5
4	8.847	305.0677	611/167/125	Gallocatechin	24.6
5	9.533	353.0887	191/179/135/161	Chlorogenic acid	54.7
6	12.100	305.0673	611/165/139/137/125	Epigallocatechin (EC)	97.2
7	12.320	353.0889	707/191/161	5-*O*-Caffeoylquinic acid (derivative)	77.9
8	12.927	353.0887	191/173/135	Cryptochlorogenic acid	109.7
9	13.493	633.0355	463/349/300	Monogalloyl-HHDP-glucose	75.1
10	13.980	471.0588	427/275/169	Epigallocatechin 3-*O*-(3′-*O*-methyl)gallate	207.6
11	15.573	337.0933	191/163/173/119	*p*-Coumaroylquinic acid	95.2
12	16.407	289.0729	245/203/151	(+)-Catechin (cinidianol)	108.6
13	16.860	457.0796	305/219/193/169	Epigallocatechin gallate (EGCG)	249.7
14	17.827	457.0791	331/305/169	Gallocatechin gallate (GCG)	47.1
15	19.053	479.0847	316/271/151	Unidentified	45.6
16	21.947	441.0894	289/245/169	(+)-Catechin gallate (CG)	182.8
17	22.213	609.0904	463/301/169	Rutin	70.4
18	24.267	593.1519	285/257/163	Kaempferol-3-rutinoside	18.6
19	27.067	447.0943	301/151/145	Quercetin rhamnoside	14.1
20	35.447	516.1395	301/125	Unidentified	LOD

* Values (μg/mL infusions) are presented as means. External standard: Rosmarinic acid (λ = 280 nm); LOQ–limit of quantification; LOD–limit of detection.

**Table 6:** Total content * (%) of tannins, hydroxycinnamic derivatives, and flavonoids in PM and GT extracts and their mixtures.

Sample	THD (%)	Total Polyphenols	Tannins	Flavonoids λ = 392 nm	Flavonoids λ = 420 nm
PM	18.9 ± 0.2	23.0 ± 0.3	16.1 ± 0.3	2.8 ± 0.1	0.6 ± 0.02
GT	4.7 ± 0.1	29.8 ± 0.2	18.8 ± 0.3	0.5 ± 0.01	0.5 ± 0.01
lyo; GT:PM	12.6 ± 0.2	26.3 ± 0.3	18.1 ± 0.1	1.0 ± 0.01	0.4 ± 0.01
mix; GT:PM	9.5 ± 0.2	27.0 ± 0.3	18.6 ± 0.4	1.1 ± 0.01	0.5 ± 0.01

* Average value in % (n = 3).

**Table 7:** Combination index range based on the log (CI) grading scale.

Interaction	CI Scale	Interpretation
Synergism	<0.1	Very strong synergism
	0.1–0.3	Strong synergism
	0.3–0.7	Synergism
	0.7–0.85	Moderate synergism
	0.85–0.90	Slight synergism
Additivity	0.90–1.10	Nearly additive
Antagonism	1.10–1.20	Slight antagonism
	1.20–1.45	Moderate antagonism
	1.45–3.3	Antagonism
	3.3–10	Strong antagonism
	>10	Very strong antagonism

## Data Availability

The data presented in this study are available upon request from the corresponding author due to privacy.

## Abbreviations

The following abbreviations are used in this manuscript:

ABTS	2,2′-azino-bis(3-ethylbenzothiazoline-6-sulfonic acid)
CI	combination index
DCF	dichlorofluorescein
DPPH	2,2-diphenyl-1-picrylhydrazyl
DRI	dose reduction index
EGCG	(-)-epigallocatechin gallate
GT	green tea
lyo; GT:PM	lyophilizate prepared from a co-macerated mixture of GT and PM
mix; GT:PM	mixture of separately lyophilized extracts GT and PM
NIH/3T3	mouse embryo fibroblast cell line clone 3T3
PM	peppermint
Q	quercetin
RA	rosmarinic acid
SDA	sequential deletion analysis
T_R_	retention time
